# Poly(ADP-Ribosyl)ation Affects Histone Acetylation and Transcription

**DOI:** 10.1371/journal.pone.0144287

**Published:** 2015-12-04

**Authors:** Loredana Verdone, Marco La Fortezza, Fabio Ciccarone, Paola Caiafa, Michele Zampieri, Micaela Caserta

**Affiliations:** 1 Istituto di Biologia e Patologia Molecolari, CNR c/o Dipartimento di Biologia e Biotecnologie, Sapienza Università di Roma, Rome, Italy; 2 Ludwig Maximilians University Munich, Planegg-Martinsried, Germany; 3 Dipartimento di Biotecnologie Cellulari ed Ematologia, Sapienza Università di Roma, Rome, Italy; 4 Istituto Pasteur-Fondazione Cenci Bolognetti, Rome, Italy; Johns Hopkins University, UNITED STATES

## Abstract

Poly(ADP-ribosyl)ation (PARylation) is a posttranslational protein modification catalyzed by members of the poly(ADP-ribose) polymerase (PARP) enzyme family. PARylation regulates a wide variety of biological processes in most eukaryotic cells including energy metabolism and cell death, maintenance of genomic stability, chromatin structure and transcription. Inside the nucleus, cross-talk between PARylation and other epigenetic modifications, such as DNA and histone methylation, was already described. In the present work, using PJ34 or ABT888 to inhibit PARP activity or over-expressing poly(ADP-ribose) glycohydrolase (PARG), we show decrease of global histone H3 and H4 acetylation. This effect is accompanied by a reduction of the steady state mRNA level of *p300*, *Pcaf*, and *Tnfα*, but not of *Dnmt1*. Chromatin immunoprecipitation (ChIP) analyses, performed at the level of the Transcription Start Site (TSS) of these four genes, reveal that changes in histone acetylation are specific for each promoter. Finally, we demonstrate an increase of global deacetylase activity in nuclear extracts from cells treated with PJ34, whereas global acetyltransferase activity is not affected, suggesting a role for PARP in the inhibition of histone deacetylases. Taken together, these results show an important link between PARylation and histone acetylation regulated transcription.

## Introduction

PARylation is a posttranslational protein modification catalyzed by enzymes belonging to the PARP family. PARPs use NAD^+^ as substrate and, upon cleaving off nicotinamide, they covalently transfer the ADP-ribosyl moiety to suitable acceptor proteins and, subsequently, elongate the chain by adding further ADP-ribose units. In this way, they are capable to modify the protein activity by creating a branched polymer, termed poly(ADP-ribose) (PAR), which can be rapidly degraded by PARG and by ADP-ribosylhydrolase 3 (ARH3) [1,2]. Free or protein-bound ADP-ribose polymers work as signal transducers by binding other proteins through their conserved PAR recognition modules, including PAR-binding motifs (PBMs), PAR-binding zinc finger (PBZF) domains, and macrodomains [3].

The founding member of the PARP family is PARP-1, also known as ADP-ribosyltransferase Diphtheria toxin-like 1 (ARTD1, [4]), a ubiquitous and abundant nuclear protein. PARP-1 catalyzes the covalent attachment of ADP-ribose polymers on itself and other acceptor proteins, including histones, DNA repair proteins, transcription factors, and chromatin modulators [5]. Initially studied in the context of DNA damage detection and repair [6,7], PARP-1 has more recently been linked to the regulation of chromatin structure and transcription [8–10]. As a structural chromatin protein, enzymatically silent PARP-1 inhibits transcription by contributing to the condensation of chromatin. However, once activated by environmental stimuli and developmental signals, PARP-1 can modify itself and other chromatin-associated proteins, thereby loosening chromatin to facilitate gene transcription [11].

The varied roles of PARP-1 in gene regulation were recently extensively reviewed [10]. Multiple mechanisms were shown to be involved. Chromatin loosening by PARP at *Drosophila* puff loci was initially observed [12]. Subsequently, PARylation of the nucleosome-remodelling ATPase ISWI was shown to inhibit its binding and chromatin condensation activity at heat shock-loci in *Drosophila* [13], while in human cells the same modification directed recruitment and activation of ALC1, a member of the SNF2 ATPase superfamily [14]. Recently, direct remodelling of nucleosomes due to histone PARylation was demonstrated [15] as well as regulation of PARP-1-dependent gene expression through promoter-directed recruitment of a nuclear NAD^+^ Synthase [16].

More importantly, cross-talk between PARP-induced modifications and other epigenetic marks was reported. Regulation of the expression and activity of the DNA methyltransferase DNMT1 by PARP-1 affected genomic DNA methylation [17,18]. PARylation of KDM5B, a histone lysine demethylase acting on trimethyl H3 lysine 4 (H3K4me3), was shown to block the binding and demethylase activity of this enzyme [19].

The link between PARP and histone acetylation, however, has received less attention. Using PJ34 or ABT888 to inhibit PARP enzymatic activity or over-expressing PARG, we observed a decrease of global histone H3 and H4 acetylation, and this effect was accompanied by a reduction in the steady state mRNA level of *p300*, *Pcaf*, and *Tnfα*, but not of *Dnmt1*. The pattern of histone H3 and H4 acetylation changes was specific for each promoter, as shown by ChIP analyses. By assaying nuclear extracts from cells treated with PJ34 for global HAT or HDAC activity, we found a regulatory role of PARylation in the inhibition of deacetylase function.

## Materials and Methods

### Cell culture and treatments

NIH3T3 mouse fibroblasts were maintained as sub-confluent culture in high-glucose Dulbecco's modified Eagle's medium, supplemented with 10% fetal bovine serum, 2 mM L-glutamine, 50 units/ml penicillin and 50 mg/ml streptomycin.

PARP inhibition was obtained by adding to the medium PJ34, 5 μM final concentration, or ABT888, 0.5 μM final concentration, for 30 min, 1 h or 3 h.

### Transfection of cells and PARG over-expression

0.5×10^6^ cells were seeded in 60×15 mm culture dishes (Greiner bio-one) and transfected with Lipofectamine Plus reagent (Invitrogen) adopting the manufacturer's protocol. Assays were performed with 4 μg/dish of purified plasmid DNA of either empty vector (pCS2) as a control or Myc–PARG construct (pCS2-Myc-PARG) or Myc–catalytically mutated PARG construct (pCS2-Myc–PARG_E757N) [20] together with 0.4 μg/dish of pBabe-puro (Addgene) vector for puromycin selection of transfected cells. After 24 hours cells were collected and whole cell extracts were prepared.

### Western blot analysis

#### Whole cell extracts

Trypsinised and phosphate-buffered saline (PBS)-washed cells were collected by centrifugation and lysed in RIPA buffer (50 mM Tris–HCl pH 8, 150 mM NaCl, 0.5% sodium deoxycholate, 0.1% SDS, 1% Nonidet P-40, 1 mM EDTA), supplemented with protease inhibitors (complete EDTA-free, Roche Applied Science). Protein concentration was determined using the Bradford protein assay reagent (Bio-Rad) with bovine serum albumin (Promega) as standard. Equal protein amounts (50 μg) were subjected to 15% or 8% SDS–PAGE and transferred to PVDF 0.2 μm (Bio-Rad) or Hybond-ECL nitrocellulose (GE Healthcare) membranes, respectively.

#### Nuclear extracts

Cells were washed twice with Phosphate-buffered saline (PBS), collected by centrifugation and treated for 30 min on ice with a nuclei specific buffer (10 mM Tris–HCl pH 7.9, 4 mM MgCl_2_, 1 mM EDTA, 0.5 mM DTT, 0.25 M sucrose, 1% Triton X-100),supplemented with protease inhibitors (complete EDTA-free, Roche Applied Science). Nuclei were collected by centrifugation at 10.000 xg at 4°C for 10 min. Pellets were lysed in RIPA bufferand processed as above.

HAT and HDAC activity were assayed using EpiQuick^TM^ kits (Epigentek), according to manufacturer instructions.

#### Antibodies

Primary antibodies used were: mouse monoclonal anti-PAR (Trevigen,4335-MC-100), mouse monoclonal anti-Myc (9E10 clone, hybridoma-conditioned medium), rat monoclonal anti-α tubulin (Santa Cruz Biotechnology, sc-53030), rabbit polyclonal anti-acetyl-histone H3, recognizing histone H3 acetylated at the N-terminus (Millipore, 06–599), rabbit polyclonal anti-acetyl-histone H4, recognizing all 4 lysine residues (Millipore, 06–866), rabbit polyclonal anti-histone H3, recognizing the C-terminal region of histone H3, (Millipore, 07–690), rabbit polyclonal anti-histone H4, recognizing the C-terminal region of histone H4 (Abcam, ab10158). Secondary antibodies used were: goat anti-mouse, anti-rabbit and anti-rat horseradish peroxidase-conjugated (Santa Cruz Biotechnology).

Detection was carried out using the ECL Western blotting detection reagents (GE Healthcare). Densitometric analysis was performed using Image Lab software (Bio-Rad). For each lane, the value corresponding to the area of histone H3 or H4 band in the membrane probed with anti-acetyl-histone H3 or H4 was normalized by dividing for the value corresponding to the area of histone H3 or H4 band in the same membrane re-probed, after mild stripping, with anti-C-terminal region of histone H3 or H4.

### qRT-PCR

Total RNA was purified by RNeasy mini kit (Qiagen). Concentration, purity and integrity of preparations were evaluatedspectrophotometrically, followed by agarose gel-ethidium bromide electrophoresis. Total RNA (1 μg) was subjected to retrotranscription using Bioscript Reverse Transcriptase (Bioline) and random hexamer mix. Amplification of cDNA was performed in triplicate for each sample, using SsoAdvanced SYBR Green supermix on a MiniOpticon Real-time PCR System (Bio-Rad). The values, obtained by three independent experiments, were normalized with *Actβ* and *Hprt*. PCR efficiency was 90–100% for each set of primers. Primers used were as follows:

*Actβ* Fw: CTTGGGTATGGAATCCTGTGGCAT;

*Actβ* Rev: GCTCAGGAGGAGCAATGATCTTGA;

*Dnmt1* Fw: GAGGACAACAAGCACAAGTTCTGC;

*Dnmt1* Rev: TGGGTATTCTCAGGCCTGTAG;

*Hprt* Fw: GTCAACGGGGGACATAAAAGT;

*Hprt* Rev: CAAAGTCTGGCCTGTATCCAA;

*p300* Fw: AGCGGCCTAAACTCTCATCTC;

*p300* Rev: GGCTGCATCTTGTACTATGCC;

*Pcaf* Fw: TGGCCAAGATGTTTCTGAACC;

*Pcaf* Rev: TTCCAAGAGCTGTCGTCTCAT;

*Tnfα* Fw: CCCCAAAGGGATGAGAAGTT;

*Tnfα* Rev: TGGGCTACAGGCTTGTCACT;

### Chromatin immunoprecipitation (ChIP)

ChIP analyses were performed on chromatin extracts using MAGnify Chromatin Immunoprecipitation System kit (Invitrogen), according to manufacturer's specifications. Cell cultures (about 1×10^6^ cells/ml) were cross-linked, in standard culture dishes, at room temperature for 10 min by formaldehyde 37% (final concentration 1%). Reaction was stopped by 5 min incubation in 0.125 M Glycine. Cell monolayer was harvested by scraping in ice-cold PBS containing protease inhibitors. After cell lysis (final concentration of cell: 10^6^ cells/50 μl) chromatin was sonicated using Bioruptor NextGen (Diagenode) to High Power, 18 cycles for 30 seconds ON, 30 seconds OFF. Average size of sonicated DNA was around 400 bp, as measured by agarose gel electrophoresis. Aliquots containing 200.000 cells were snap-freezed and stored at -80°C. Sheared chromatin was immunoprecipitated with anti-acetyl-Histone H3 or anti-acetyl-Histone H4, or rabbit IgG as negative control. DNA amplification was performed using SsoAdvanced SYBR Green supermix on a MiniOpticon Real-time PCR System (Bio-Rad).

The Ct values for each gene promoter, obtained from three biological replicates of samples analysed in triplicate, were normalized with an internal region of *Actβ* and INPUT DNA, as follows: first, the Ct value of the immunoprecipitated (IP) target gene was corrected subtracting the Ct value of the *Actβ* IP; then, the Ct value of the target gene INPUT was corrected subtracting the Ct value of the *Actβ* INPUT; finally, the normalized target gene IP value was corrected subtracting the normalized INPUT value.

Primers used were as follows:

*Actβ* Fw: AAGCATCCTTAGCTTGGTGAG, Rev: ACAAGATGGTGAATGGTGAG (spanning region from +2666 to + 2769)

*Dnmt1* A1 Fw: TATAGCCAGGAGGTGTGGGTG, Rev: AACGAGACCCCGGCTTTTT (spanning region from -2 to +160);

*Dnmt1* A2 Fw: TCCTCTGCAAGAGCAGCACTA, Rev: ATGTACCACACAGGGCAAGA (spanning region from -100 to +93);

*p300* Fw: AGCTCAGTGTGGCCATTAGG, Rev: TGTCCTCCTCCTTCTCATCG (spanning region from -183 to +13);

*Pcaf* Fw: ACGCCATGATTTTGGTGAAT, Rev: GAGACCCAACTTCCTCCACC (spanning region from -107 to +110);

*Tnfα* Fw: GTTTTCCGAGGGTTGAATGAG, Rev: TCTGTTCTCCCTCCTGGCTA (spanning region from -79 to +54).

A map describing the position of the promoter fragments analysed for each target gene is presented in S2 Fig.

### Statistical analysis

Statistical analysis was carried out by the Student's *t*-test. Differences were considered significant when p≤0.05.

## Results

### Global decrease of histone H3 and H4 acetylation level following PARP inhibition

**Fig 1:**
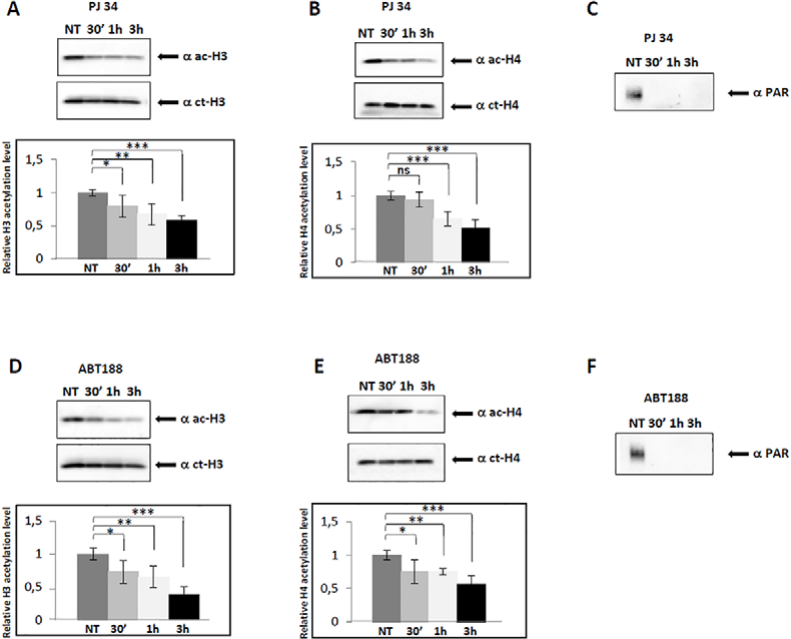
PJ34- and ABT888-induced reduction of PARs influences histone H3 and H4 acetylation. (A) Representative Western blot of whole cell extracts from cells treated with the inhibitor PJ34 (5μM) at the indicated times relative to untreated cells (NT, value ~1), run on a 15% SDS-PAGE, and probed with anti-acetyl-histone H3 and anti-C-terminal of H3 antibodies to measure the relative level of H3 acetylation. Error bars indicate the standard deviation of data obtained from three independent experiments. (B) Same as in (A) but hybridisation was performed with anti-acetyl-histone H4 and anti-C-terminal of H4 antibodies to measure the relative level of H4 acetylation. (C) The same extracts used in panels A and B were run on an 8% SDS-PAGE and probed with anti-PAR antibody to visualize ADP-ribose polymers level. (D) Same as in (A) but from cells treated with the inhibitor ABT888 (0.5 μM). (E) Same as in (D) but hybridisation was performed with anti-acetyl-histone H4 and anti-C-terminal of H4 antibodies to measure the relative level of H4 acetylation. (F) The same extracts used in panels D and E were run on an 8% SDS-PAGE and probed with anti-PAR antibody to visualize PARs level. *p ≤ 0.05; **p ≤ 0.01; ***p ≤ 0.001

To determine the influence of PARP activity on the global histone acetylation level, we initially analysed by western blot the extent of both histones H3 and H4 lysine acetylation in conditions in which PARP enzymes are inhibited by PJ34. Mouse NIH3T3 cells were treated with PJ34 for 30 min, 1 hand 3 h. Whole cell extracts were prepared, and aliquots were run on SDS-PAGE gels, transferred to a membrane and probed with antibodies recognizing acetylated lysines of histone H3 and H4 tails. The results are shown in Fig 1, panels A and B for H3 and H4, respectively. Following inhibition of PARPs activity with PJ34, we observed a decrease of lysine acetylation in both histone H3 and H4 tails, within 3 h treatment. As a control for reduced level of ADP-ribose polymers, we utilised on the same cell extracts an antibody against PAR (Fig 1, panel C). In order to exclude a non specific effect of the drug utilized to inhibit PARPs, we repeated the same experiment with the inhibitor ABT888, and the results are shown in Fig 1, panels D and E for H3 and H4, respectively. PARPs inhibition by ABT888, as shown by decreased PAR levels (Fig 1, panel F), leads to decreased H3 and H4 acetylation. Taken together, the results obtained with the two different inhibitors underline the true involvement of PARP activity rather than reflecting the effect of the drug utilized.

Moreover, the decrease of histone H3 and H4 acetylation level, following PARP inhibition, was not a peculiarity of NIH3T3 cells, because it was observed also in another mouse fibroblast cell line, L929, as well as in the neuroblastoma cell type N2a (see S4 Fig).

### PARG over-expression induces global decrease of histone H3 and H4 acetylation

**Fig 2:**
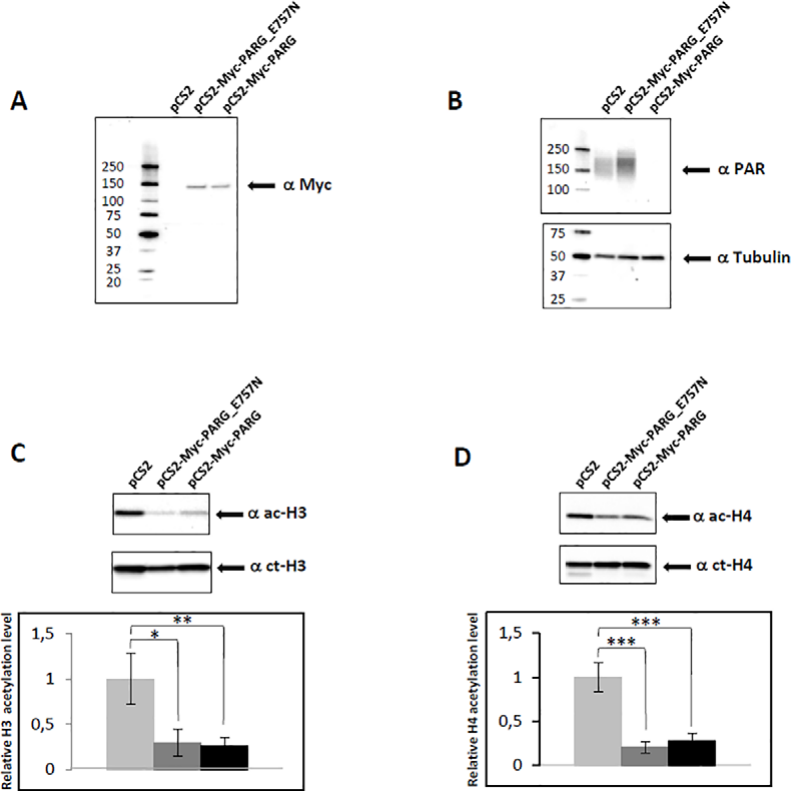
Ectopic over-expression of PARG influences histone acetylation levels. (A) Representative Western blot of total proteins from cells transfected with empty vector (pCS2) or Myc–catalytically mutated PARG construct (pCS2-Myc-PARG_E757N) or Myc–PARG construct (pCS2-Myc-PARG) run on an 8% SDS-PAGE, and probed with anti-Myc epitope to check for exogenous protein expression. (B) Same as in (A) but hybridisation was performed with anti-PAR antibody to visualize PARs level, and with anti-α Tubulin as protein loading control. (C) The same extracts used in panel A were run on a 15% SDS-PAGE, and probed with anti-acetyl-histone H3 and anti-C-terminal of H3 antibodies. Histograms indicate acetylation level of cells transfected with pCS2-Myc-PARG construct (black), or with pCS2-Myc-PARG_E757N (grey) relative to control cells transfected with empty vector (white, value ~1). Error bars indicate the standard deviation of data obtained from three independent experiments. (D) As in (C), but using anti-acetyl-histone H4 and anti-C-terminal of H4 antibodies to measure the relative level of H4 acetylation. *p ≤ 0.05; **p ≤ 0.01; ***p ≤ 0.001

To investigate the molecular mechanism underlying the global decrease of histone tail acetylation, observed in condition of inhibited PARP activity, we sought to analyse whether the same effect can be observed by reducing PAR through a different approach. In order to deplete the cells of PAR, we ectopically over-expressed the PARG enzyme. Whole cells extracts were prepared after transfection with the empty vector pCS2 or with Myc-PARG constructs, one over-expressing PARG and one containing a catalytically mutated PARG. We checked for exogenous protein expression by western blotting, as reported in Fig 2, panel A. Panel B shows that in cells containing plasmid pCS2-Myc-PARG no anti-PAR signal is visible, while the usual smear of PAR is present in cells containing the empty vector. In the extracts from cells containing plasmid pCS2-Myc-PARG, we observed reduced level of histone H3 and H4 tails acetylation (panel C and D, respectively), relative to the control. Interestingly, in the extracts from cells transfected with the plasmid containing catalytically mutated PARG, pCS2-Myc-PARG_E757N [20], decreased levels of histone H3 and H4 acetylation were observed (panel C and D, respectively), even though these cells are characterized by a greater amount of PAR (panel B).

In summary, the observed decrease of lysine acetylation, for both histone H3 and H4, is due to an unbalance in the level of PAR (see Discussion).

### PARP inhibition down-regulates *p300* and *Pcaf* expression by decreasing promoter histone H3 and H4 acetylation

**Fig 3:**
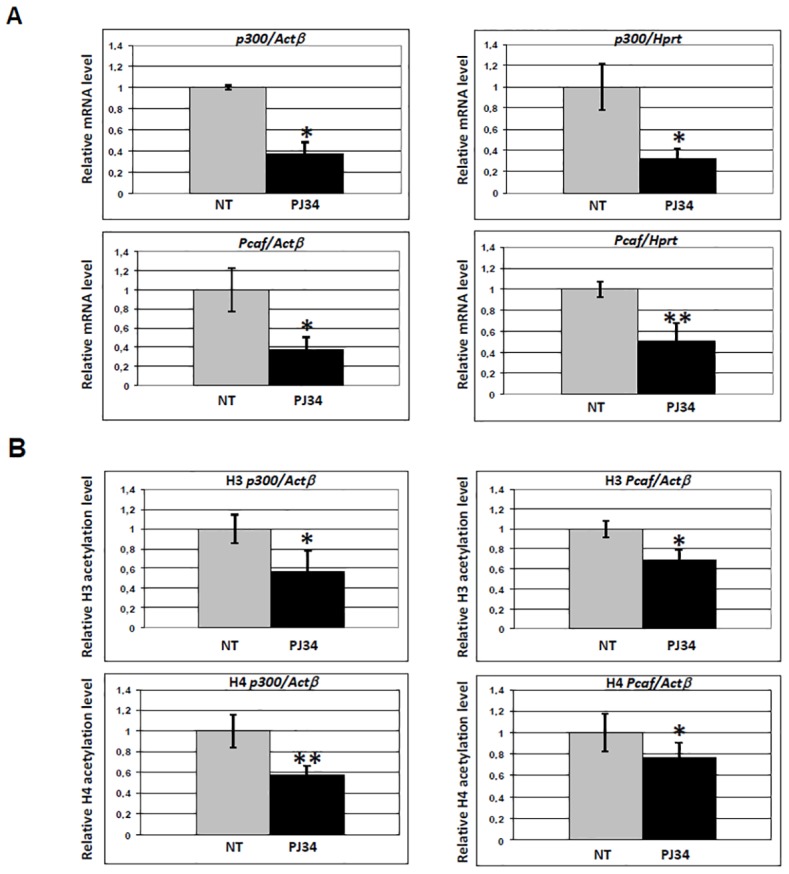
Inhibition of PARP activity affects transcription and promoter histone acetylation level of *p300* and *PCAF*. (A) Real time qRT-PCR analysis of mRNA accumulation for two acetyltransferases, *p300* and *Pcaf*. Histograms indicate mRNA level of cells treated with PJ34 for 1 h (black), relative to untreated cells (grey, value ~1). Values were normalized with *Actβ* or *Hprt*. (B) Real time qRT-PCR analysis of histone H3 and H4 acetylation of *p300* and *Pcaf* promoter regions. Histograms indicate acetylation level of cells treated with PJ34 for 1 h (black), relative to untreated cells (grey, value ~1). Values were normalized with an internal region of *Actβ*. Primers used were described in Materials and Methods. Error bars indicate the standard deviation of data obtained from three independent experiments. NT: control cells, no treatment. *p ≤ 0.05; **p ≤ 0.01.

In order to investigate the link between histone acetylation and PARP activity, we next analysed the mRNA steady state level of the genes coding for two relevant enzymes responsible for the maintenance of histone acetylation, namely *p300* and *Pcaf* by qRT-PCR analysis. As shown in Fig 3A, significant down-regulation of both genes was detected after 1 h of treatment with PJ34. However, the transcriptional effect observed at 1 h did not lead to decreased protein amount up to 3 h (S1 Fig), suggesting that the global H3 and H4 acetylation decrease reported was not due to reduced amount of p300 and PCAF. Rather, reduced acetyltransferase activity or increased deacetylase activity could have been involved.

We hypothesized that hypoacetylation in the chromatin region spanning the TSS was responsible for decreased *p300* and *Pcaf* mRNA accumulation. Therefore, we investigated by ChIP the promoter region of *p300* and *Pcaf* genes, by using the same antibodies utilized in the western blot experiments, and the results are shown in Fig 3B. Acetylation of both histone H3 and H4 significantly decreased following 1 h of PARP inhibition.

We conclude that PARP activity promotes *p300* and *Pcaf* transcription by maintaining the correct level of histone H3 and H4 acetylation at their TSS.

### PARP inhibition leads to decreased histone H3 but not histone H4 acetylation at the *Dnmt1* promoter

In order to better investigate the relationship between promoter acetylation and PARP activity we tested by ChIP assay the *Dnmt1* gene, coding for DNA methyltransferase 1, because the expression and the activity of this enzyme are known to be regulated by PARP-1 [17,21]. Since it was previously shown that the *Dnmt1* promoter is occupied by poly(ADP-ribosyl)ated PARP-1, and that the decrease of PAR leads to aberrant methylation of the CpG island close to the TSS [22], we searched for the possible involvement of histone acetylation.

**Fig 4:**
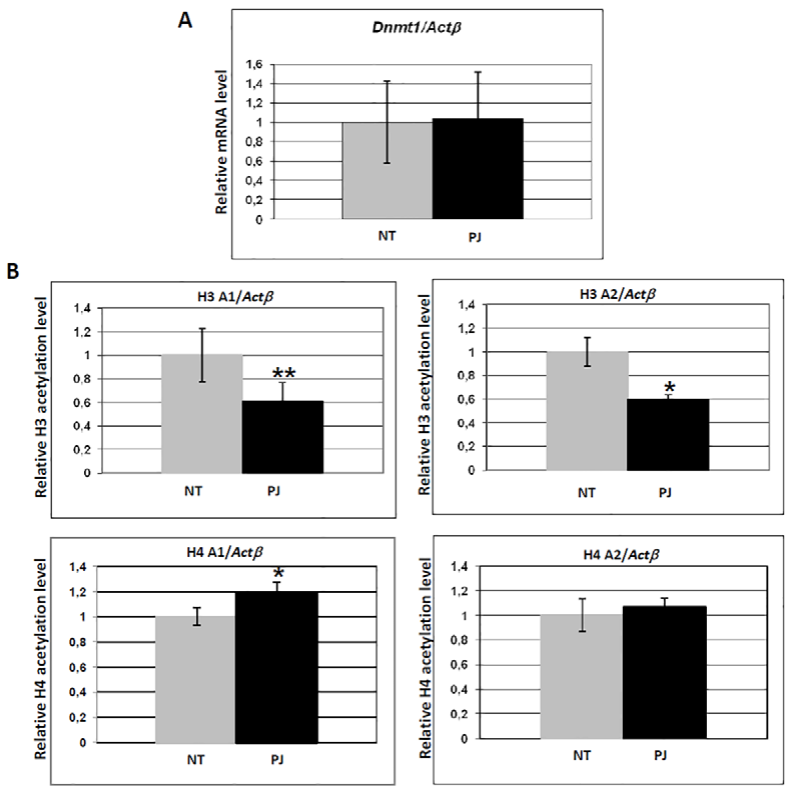
Inhibition of PARP activity does not affect transcription of *Dnmt1*, but influences its promoter acetylation level. (A) Real time qRT-PCR analysis of mRNA accumulation for *Dnmt1*. Histogram indicates mRNA level of cells treated with PJ34 for 1 h (black), relative to untreated cells (grey, value ~1). Values were normalized with *Actβ*. (B) Real time qRT-PCR analysis of histone H3 and H4 acetylation of two *Dnmt1* promoter regions. Histograms indicate acetylation level ofcells treated with PJ34 for 1 h (black), relative to untreated cells (grey, value ~1). Values were normalized with an internal region of *Actβ*. Primers A1 and A2 were described in Materials and Methods. Error bars indicate the standard deviation of data obtained from three independent experiments. NT: control cells, no treatment. *p ≤ 0.05; **p ≤ 0.01.

After PJ34 treatment for 1 h, a decrease of histone H3 acetylation at the *Dnmt1* promoter region spanning the TSS was observed, while histone H4 acetylation was slightly increased. This pattern of histone tails acetylation changes did not affect *Dnmt1* transcription (Fig 4).

### PARP inhibition down-regulates basal *Tnfα* transcription

**Fig 5:**
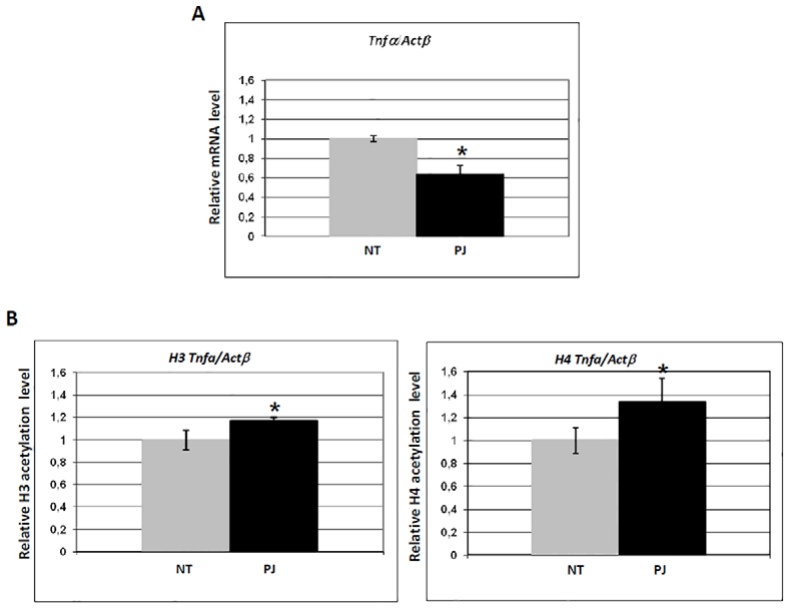
PARP inhibition down-regulates constitutive *Tnfα* transcription. (A) Real time qRT-PCR analysis of mRNA accumulation for *Tnfα*. Histogram indicates mRNA level of cells treated with PJ34 for 1 h (black), relative to untreated cells (grey, value ~1). Values were normalized with *Actβ*. (B) Real time qRT-PCR analysis of histone H3 and H4 acetylation of *Tnfα* promoter region. Histograms indicate acetylation level of cells treated with PJ34 for 1 h (black), relative to untreated cells (grey, value ~1). Values were normalized with an internal region of *Actβ*. Error bars indicate the standard deviation of data obtained from three independent experiments. NT: control cells, no treatment. *p ≤ 0.05.

We then investigated if the mRNA accumulation of other genes known to be PARP-1-dependent was affected by PARP inhibition. In particular, we tested the *Tnfα* promoter, known to be involved in the inflammatory response to antigenic stimuli, and whose activation was shown to be impaired in *PARP-1* knockout mice [23]. Cells were treated with PJ34 for 1 h, and the results are shown in Fig 5A: *Tnfα* basal transcription was affected by PARP inhibition, as indicated by significant decrease of mRNA accumulation. When the TSS region of the *Tnfα* promoter was investigated by ChIP after PARP inhibition for 1h, using antibodies against histone H3 or H4 acetylation, a slight but significant increase in histone acetylation levels was observed (see Fig 5B), suggesting that, in this case, PARylation is regulating transcription through a mechanism other than acetylation of H3 or H4 (see Discussion).

### PARP inhibition up-regulates global HDAC activity

**Fig 6:**
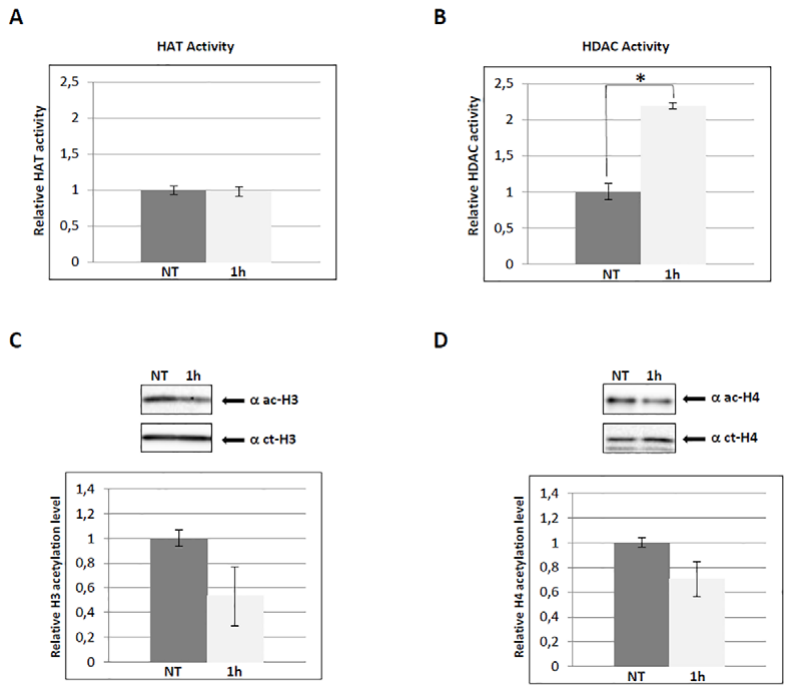
PARP inhibition up-regulates global HDAC activity. (A) Nuclear extracts from NIH3T3 cells, treated with the inhibitor PJ34 (5 μM) for 1 h, and assayed for global HAT activity. Histograms indicate colorimetric quantification, through an ELISA-like reaction, of HAT activity in treated cells relative to untreated (NT, value ~1). Error bars indicate the standard deviation of data obtained from three independent experiments. (B) Same as in (A) but the assay was for global HDAC activity. *p ≤ 0.05. (C) The same nuclear extracts as in (A) and (B) were run on a 15% SDS-PAGE, and probed with anti-acetyl-histone H3 and anti-C-terminal of H3 antibodies to measure the relative level of H3 acetylation. Error bars indicate the standard deviation of data obtained from three independent experiments. (D) Same as in (C) but hybridisation was performed with anti-acetyl-histone H4 and anti-C-terminal of H4 antibodies to measure the relative level of H4 acetylation.

In order to gain a better insight on the molecular mechanism responsible for the observed effects, we tested nuclear extracts from NIH3T3 cells, treated with PJ34 for 1h, for global acetyltransferase or deacetylase activity, and the results are shown in Fig 6. Global deacetylase activity was found to increase upon PARPs inhibition (panel B), whereas acetyltransferase activity did not change when tested in the same conditions (panel A). Panel C shows the decrease of histone H3 and H4 acetylation in the same nuclear extracts, as expected.

These data suggest that in normal conditions PARP activity is required to prevent default histone deacetylation, thus allowing maintenance of constitutive transcription.

## Discussion

Eukaryotic gene regulation occurs at many steps during the process of gene expression: chromatin structure is one of the key control step. External stimuli are capable of modifying the epigenetic code associated with a specific promoter to allow for transcription and cell adaptation. This concept is easily demonstrated in simple organisms such as yeast where the availability of mutants allows the association of specific chromatin patterns to defined phenotypes under different environmental conditions. In more complex systems, depletion of proteins involved in epigenetic control, as well as the use of drugs capable of altering their activity are becoming increasingly useful: in addition to provide evidence of changes at the level of the specific mark, they could also shed light on the possible interplay among epigenetic marks [24–27].

Cross-talk between PARylation and other epigenetic marks was reported. Regulation of the activity of the DNA methyltransferase DNMT1 by PARP-1 affected genomic DNA methylation [17,18]. PARylation of KDM5B, a histone lysine demethylase acting on trimethyl H3 lysine 4 (H3K4me3), was shown to prevent the binding and demethylase activity of this enzyme [19]. The connection between PARP activity and histone acetylation, however, has received less attention. Earlier *in vitro* reports suggested a correlation between the two posttranslational modification processes [28,29], while subsequent evidence points to a competition between them [30,31]. In cultured cortical neurons histone H4 acetylation was shown to be affected by PARP-1 activation [32]. More recently, an interplay between histone acetylation/deacetylation and poly(ADP-ribosyl)ation was shown [33–34]. In order to investigate the interdependence between PARylation and histone tail acetylation, we studied the effect of PARP inhibition both on global histone acetylation level and at specific promoters. We chose to utilize antibodies recognizing all acetylated lysines at the same time on either H3 or H4, instead of antibodies against single lysines, to get a general idea about the hypothesized correlation. We show that the global levels of histone H3 and H4 acetylation decrease as a consequence of alteration of PARP function by two different strategies: i) using PJ34 or ABT888 to inhibit PARP enzymatic activity (Fig 1); ii) over-expressing PARG to deplete the cell of ADP-ribose polymers (Fig 2). Moreover, we utilised a third experimental strategy, *i*.*e*., over-expression of a catalytically mutated PARG, to analyse the effect of increasing the amount of PARs relative to a normal condition, and we show that even in this case histone H3 and H4 acetylation decreases. Since we found that inhibition of PARylation caused an increase in global HDAC activity (Fig 6), we suggest that PARs reduction, due to PARP inhibitors or PARG over-expression lead to failed inhibition of HDACs, thus explaining histone acetylation decrease. To explain why the same effect is observed also in the presence of higher amount of PARs, present in cells transfected with the catalytically mutated PARG, we hypothesize that excess PARylation modifies PARP-1, causing it to be released from chromatin [35]. Presumably, chromatin bound PARP-1 is required to interact with remodelling factors on gene promoters and to recruit/regulate epigenetic marks. Thus, poly(ADP-ribose) levels that are either too high or too low lead to the same outcome in terms of global histone acetylation state: they both cause widespread histone hypoacetylation. These results underscore the critical importance of keeping balanced PAR levels, as already argued relatively to the cross-talk between poly(ADP-ribosyl)ation and DNA methylation [18]. When we used an alternative strategy to increase ADP-ribose polymers level, *i*.*e*., by over-expressing the architectural protein CTCF (CCCTC-binding factor), which strongly stimulates PARP-1 activity [36], we again observed global decrease of H3 acetylation (S5 Fig). In addition, we analysed extracts of cells treated with the PARG inhibitor gallotannin, and found a transient increase of histone H3 and H4 acetylation at 30 min, followed by a decrease within 3 h of treatment (S6 Fig). The transient increase observed was possibly due to inhibition of global HDAC activity by increased PARs.

It has been shown that PARP-1 and PARG are localized at the promoters of positively and negatively regulated target genes, where they act in concert to control the amount of PARs [8].Based on the results obtained by assaying HAT and HDAC activity upon PARP inhibition (Fig 6), we hypothesize that, in normal conditions, ADP-ribose polymers regulate histone lysine acetylation and transcription by inhibiting the function of deacetylases. PARP-1 was indeed described in complex with HDACs 1–3, but not HDACs 4–6, to inhibit their function [37]. Other nuclear PARPs could be implicated as well: PARP-14 was shown to interact with HDAC2 and HDAC3 [38]. We cannot exclude that, in defined conditions, ADP-ribose polymers could affect also the function of acetyltransferases. For example, PARP-1 was found in complex with p300 [39], suggesting a stimulatory effect on this enzyme.

Besides global effects, we described alteration of specific regulatory patterns at the level of defined promoters, as follows: i) PJ34-induced PARP inhibition leads to decreased acetylation at both histone H3 and H4 around the TSS region of *p300* and *Pcaf* promoters (Fig 3B), and this effect correlates with decreased mRNA accumulation: histone tails acetylation is, in fact, responsible for keeping promoter nucleosomes accessible ([40] and ref therein); ii) in the case of the *Dnmt1* promoter, only H3 acetylation decreases, while histone H4 acetylation is slightly increased (Fig 4): this pattern of modifications does not parallel transcriptional down-regulation, suggesting a compensatory role for H4 over H3 acetylation, as proposed in the case of *S*. *cerevisiae* Adr1-regulated genes [41]; iii) *Tnfα* constitutive transcription is affected by PARP inhibition, as indicated by significant decrease of basal mRNA accumulation after PJ34 treatment (Fig 5A). When the TSS region of the *Tnfα* promoter is investigated by ChIP in non-inducing conditions, a slight but significant increase of histone H3 and H4 acetylation is observed (Fig 5B), suggesting that, in this specific case, PARylation is likely regulating transcription through a mechanism other than acetylation of H3 or H4. For example, PARylation of histones could be implicated [31,42–44]: since the acceptor sites for this modification are lysine residues, the possibility exists that PJ34 induced PAR reduction allows for the observed acetylation increase. An alternative explanation can be provided when considering that histone acetylation was found to be in continuous dynamic turnover [45,46]. When this turnover was inhibited, the expression of certain genes was no longer possible, despite the increase of histone acetylation levels at their promoters [45,46]. This indicates that cycles of histone acetylation and deacetylation are important for induction of certain genes in mammalian cells.

We argue that for each promoter different patterns of interactions between PARPs and histone acetyltransferases/deacetylases and chromatin remodelers occur.

Taken together, our results indicate that PARP activity affects transcription by modulating histone H3 and H4 acetylation at the level of specific promoters in a context-dependent manner, thus underlying gene- and cell type-specific effects in determining PARP-dependent outcomes, as already suggested [8,10].

The data presented in this work could help to address future investigations aimed at identifying the specific targets of PARP activity among the enzymes regulating histone acetylation, in particular deacetylases. In addition, it will be important to identify the specificity of acetylation/deacetylation sites and how are they regulated.

## Supporting Information

S1 Fig Analysis of acetyltransferase protein level. Western blot analysis of p300 (A) and PCAF (B) in extracts from cells treated with PJ34 for 3 h relative to untreated cells (NT). Histograms indicate p300 and PCAF protein level, normalized with α tubulin, from cells treated with PJ34 for 3 h (black) relative to untreated cells (NT, grey). Antibodies used were: mouse monoclonal anti-KAT3B/p300 CT (Millipore, 05–257), rabbit polyclonal anti-KAT2B/PCAF (abcam, ab12188), and rat monoclonal antibody anti-α tubulin (Santa Cruz Biotechnology, sc-53030).
(TIF)

S2 Fig Map of the promoter fragments used in ChIP assays. For each gene the TSS (Transcriptional Start Site) and the ATG are indicated.
(TIF)

S3 Fig Analysis of PARG expression. (A) qRT-PCR of *Parg* mRNA level from NIH3T3 cells treated with PJ34 for the indicated times, relative to untreated cells. The mRNA values were normalised to the mean expression of two housekeeping genes, *Gusb* and *Hprt1*. Taqman probes were: Mm00449466_m1 for *Parg*, Mm00446956_m1 for *Gusb*, and Mm00446968_m1 for *Hprt1*. qRT-PCR conditions were as described in Ciccarone et al., Plos One 2008. Error bars indicate the standard deviation of data obtained from three independent experiments. (B) Western blot of whole cell extracts from the same cells as in (A) run on an 8% SDS-PAGE, and probed with anti-PARG (Santa Cruz sc-21480), or anti-PAR, or anti-Tubulin antibodies.
(TIF)

S4 Fig Analysis of global histone acetylation level in L929 and N2a cells. (A) Western blot of whole cell extracts from L929 cells treated with the inhibitor PJ34 (5 M) for 3 h relative to untreated cells (NT, value ~1), run on a 15% SDS-PAGE, probed with anti-acetyl-histone H3 or anti-C-terminal of H3 antibodies to measure the relative level of H3 acetylation. Error bars indicate the standard deviation of data obtained from the three independent experiments. (B) Same as in (A) but hybridisation was performed with anti-acetyl-histone H4 or anti-C-terminal of H4 antibodies to measure the relative level of H4 acetylation. (C) The same extracts used in panels A and B were run on an 8% SDS-PAGE and probed with anti-PAR antibody to visualize ADP-ribose polymers level. Panels D, E and F: same as in A, B and C, respectively but the analysis was done with whole cell extracts from neuroblastoma N2a cells.
(TIF)

S5 Fig Analysis of global histone H3 acetylation level upon CTCF overexpression. (A) Western blot of total proteins from cells transfected with empty vector (pCI) or His-tagged recombinant CTCF (pCI-CTCF-His, Guastafierro et al., J. Biol. Chem 2008) run on an 8% SDS-PAGE, and probed with anti-PAR antibody. (B) Same as in (A) but hybridisation was performed with anti-His antibody to visualize CTCF, and with anti-Lamin B1, as protein loading control. (C) The same extracts used in panel A and B were run on a 15% SDS-PAGE, and probed with anti-acetyl-histone H3 and anti-C-terminal of H3 antibodies.
(TIF)

S6 Fig Analysis of global histone acetylation level in the presence of gallotannin. (A) Western blot of whole cell extracts from NIH 3T3 cells treated with the PARG inhibitor gallotannin (Tannic Acid, Sigma) (30 M) for the indicated times relative to untreated cells (NT) run on an 8% SDS-PAGE, and probed with anti-PAR antibody to visualize ADP-ribose polymers. (B) The same extracts as in (A) were run on a 15% SDS-PAGE and probed with anti-acetyl-histone H3 or anti-C-terminal of H3 antibodies to measure the relative level of H3 acetylation. (C) Same as in (B) but hybridisation was performed with anti-acetyl-histone H4 or anti-C-terminal of H4 antibodies to measure the relative level of H4 acetylation.
(TIF)

## Abbreviations

PARylation: Poly(ADP-ribosyl)ation
PARP: Poly(ADP-ribose) polymerase
PARG: poly(ADP-ribose) glycohydrolase
PAR: poly(ADP-ribose)
ChIP: chromatin immunoprecipitation
qRT-PCR: (quantitative Reverse Transcription Polymerase Chain Reaction)
TSS: Transcription Start Site
HAT: histone acetyl transferase
HDAC: histone deacetylase

## References

[1] Hassa PO, Hottiger MO. The diverse biological roles of mammalian PARPS, a small but powerful family of poly-ADP-ribose polymerases. Front Biosci. 2008 1 1;13:3046–82. 17981777 10.2741/2909

[2] Bürkle A, Virág L. Poly(ADP-ribose): PARadigms and PARadoxes. Mol Aspects Med. 2013 12; 34(6):1046–65. 10.1016/j.mam.2012.12.010 Epub 2013 Jan 2. 23290998

[3] Kraus WL. New functions for an ancient domain. Nat Struct Mol Biol. 2009 9; 16(9):904–7. 19739287 10.1038/nsmb0909-904

[4] Hottiger MO, Hassa PO, Lüscher B, Schüler H, Koch-Nolte F. Toward a unified nomenclature for mammalian ADP-ribosyltransferases. Trends Biochem Sci. 2010 4; 35(4):208–19. 10.1016/j.tibs.2009.12.003 Epub 2010 Jan 26. 20106667

[5] Luo X, Kraus WL. On PAR with PARP: cellular stress signaling through poly(ADP-ribose) and PARP-1. Genes Dev. 2012 3 1; 26(5):417–32. 10.1101/gad.183509.111 22391446 PMC3305980

[6] Malanga M, Althaus FR. The role of poly(ADP-ribose) in the DNA damage signaling network. Biochem Cell Biol. 2005 6; 83(3):354–64. 15959561 10.1139/o05-038

[7] Ahel D, Horejsí Z, Wiechens N, Polo SE, Garcia-Wilson E, Ahel I, et al Poly(ADP-ribose)-dependent regulation of DNA repair by the chromatin remodeling enzyme ALC1. Science. 2009 9 4; 325(5945):1240–3. 10.1126/science.1177321 Epub 2009 Aug 6. 19661379 PMC3443743

[8] Frizzell KM, Gamble MJ, Berrocal JG, Zhang T, Krishnakumar R, Cen Y, et al Global analysis of transcriptional regulation by poly(ADP-ribose) polymerase-1 and poly(ADP-ribose) glycohydrolase in MCF-7 human breast cancer cells. J Biol Chem. 2009 12 4; 284(49):33926–38. 10.1074/jbc.M109.023879 Epub 2009 Oct 7. 19812418 PMC2797163

[9] Krishnakumar R, Kraus WL. The PARP side of the nucleus: molecular actions, physiological outcomes, and clinical targets. Mol Cell. 2010 7 9; 39(1):8–24. 10.1016/j.molcel.2010.06.017 20603072 PMC2923840

[10] Kraus WL, Hottiger MO. PARP-1 and gene regulation: progress and puzzles. Mol Aspects Med. 2013 12; 34(6):1109–23. 10.1016/j.mam.2013.01.005 Epub 2013 Jan 26. 23357755

[11] Ji Y, Tulin AV. The roles of PARP1 in gene control and cell differentiation. Curr Opin Genet Dev. 2010 10; 20(5):512–8. 10.1016/j.gde.2010.06.001 Epub 2010 Jun 28. 20591646 PMC2942995

[12] Tulin A, Spradling A. Chromatin loosening by poly(ADP)-ribose polymerase (PARP) at Drosophila puff loci. Science. 2003 1 24; 299(5606):560–2. 12543974 10.1126/science.1078764

[13] Sala A, La Rocca G, Burgio G, Kotova E, Di Gesù D, Collesano M, et al The nucleosome-remodeling ATPase ISWI is regulated by poly-ADP-ribosylation. PLoS Biol. 2008 10 14; 6(10):e252 10.1371/journal.pbio.0060252 18922045 PMC2567001

[14] Gottschalk AJ, Timinszky G, Kong SE, Jin J, Cai Y, Swanson SK, et al Poly(ADP-ribosyl)ation directs recruitment and activation of an ATP-dependent chromatin remodeler. Proc Natl Acad Sci U S A. 2009 8 18; 106(33):13770–4. 10.1073/pnas.0906920106 Epub 2009 Aug 6. 19666485 PMC2722505

[15] Martinez-Zamudio R, Ha HC. Histone ADP-ribosylation facilitates gene transcription by directly remodeling nucleosomes. Mol Cell Biol. 2012 7; 32(13):2490–502. 10.1128/MCB.06667-11 Epub 2012 Apr 30. 22547677 PMC3434492

[16] Zhang T, Berrocal JG, Yao J, DuMond ME, Krishnakumar R, Ruhl DD, et al Regulation of poly(ADP-ribose) polymerase-1-dependent gene expression through promoter-directed recruitment of a nuclear NAD+ synthase. J Biol Chem. 2012 4 6; 287(15):12405–16. 10.1074/jbc.M111.304469 Epub 2012 Feb 13. 22334709 PMC3320990

[17] Reale A, Matteis GD, Galleazzi G, Zampieri M, Caiafa P. Modulation of DNMT1 activity by ADP-ribose polymers. Oncogene. 2005 1 6; 24(1):13–9. 15637587 10.1038/sj.onc.1208005

[18] Caiafa P, Guastafierro T, Zampieri M. Epigenetics: poly(ADP-ribosyl)ation of PARP-1 regulates genomic methylation patterns. FASEB J. 2009 3; 23(3):672–8. 10.1096/fj.08-123265 Epub 2008 Nov 11. 19001527

[19] Krishnakumar R, Kraus WL. PARP-1 regulates chromatin structure and transcription through a KDM5B-dependent pathway. Mol Cell. 2010 9 10; 39(5):736–49. 10.1016/j.molcel.2010.08.014 20832725 PMC2939044

[20] Guastafierro T, Catizone A, Calabrese R, Zampieri M, Martella O, Bacalini MG, et al ADP-ribose polymer depletion leads to nuclear Ctcf re-localization and chromatin rearrangement. Biochem J. 2013 2 1; 449(3):623–30. 10.1042/BJ20121429 23116180

[21] Zardo G, Reale A, Passananti C, Pradhan S, Buontempo S, De Matteis G, et al Inhibition of poly(ADP-ribosyl)ation induces DNA hypermethylation: a possible molecular mechanism. FASEB J. 2002; 16:1319–1321. 12154007 10.1096/fj.01-0827fje

[22] Zampieri M, Passananti C, Calabrese R, Perilli M, Corbi N, De Cave F, et al PARP-1 localizes within the Dnmt1 promoter and protects its unmethylated state by its enzymatic activity. PLoS One 2009; 4: e4717 10.1371/journal.pone.0004717 19262751 PMC2650799

[23] Altmeyer M, Hottiger MO. Poly(ADP-ribose) polymerase 1 at the crossroad of metabolic stress and inflammation in aging. Aging (Albany NY) 2009; 1:458–469. 20157531 10.18632/aging.100052 PMC2806023

[24] Murr R. Interplay between different epigenetic modifications and mechanisms. Adv. Genet. 2010; 70:101–141. 10.1016/B978-0-12-380866-0.60005-8 20920747

[25] Bartke T, Kouzarides T. Decoding the chromatin modification landscape. Cell Cycle 2011; 10:182 10.4161/cc.10.2.14477 21224725

[26] Du J, Patel DJ. Structural biology-based insights into combinatorial readout and cross-talk among epigenetic marks. Biochim. Biophys. Acta 2014; 1839:719–727 2014. 10.1016/j.bbagrm.2014.04.011 24747177 PMC4689310

[27] Rothbart SB, Strahl BD. Interpreting the language of histone and DNA modifications. Biochim. Biophys. Acta 2014; 1839:627–643. 10.1016/j.bbagrm.2014.03.001 24631868 PMC4099259

[28] Malik N, Smulson M. A relationship between nuclear poly(adenosine diphosphate ribosylation) and acetylation posttranslational modifications. 1. Nucleosome studies. Biochemistry 1984; 23:3721–3725. 6089879 10.1021/bi00311a023

[29] Wong M, Smulson M. A relationship between nuclear poly(adenosine diphosphate ribosylation) and acetylation posttranslational modifications. 2. Histone studies. Biochemistry 1984; 23:3726–3730. 6477891 10.1021/bi00311a024

[30] Boulikas T. Poly(ADP-ribosylated) histones in chromatin replication. J. Biol. Chem. 1990; 265:14638–14647. 2387872

[31] Messner S, Altmeyer M, Zhao H, Pozivil A, Roschitzki B, Gehrig P, et al PARP-1 ADP-ribosylates lysine residues of the core histone tails. Nucleic Acids Res. 2010; 38:6350–6362. 10.1093/nar/gkq463 20525793 PMC2965223

[32] Cohen-Armon M, Visochek L, Rozensal D, Kalal A, Geistrikh I, Klein R, et al DNA-independent PARP-1 activation by phosphorylated ERK2 increases Elk1 activity: a link to histone acetylation. Mol. Cell 2007; 25:297–308. 10.1016/j.molcel.2006.12.012 17244536

[33] Yang Z, Li L, Chen L, Yuan W, Dong L, Zhang Y, et al PARP-1 mediates LPS-induced HMGB1 release by macrophages through regulation of HMGB1 acetylation. J Immunol. 2014; 193:6114–6123. 10.4049/jimmunol.1400359 25392528

[34] Gerace E, Landucci E, Scartabelli T, Moroni F, Chiarugi A, Pellegrini-Giampietro DE. Interplay between histone acetylation/deacetylation and poly(ADP-ribosyl)ation in the development of ischemic tolerance in vitro. Neuropharmacology. 2015; 92:125–134. 10.1016/j.neuropharm.2015.01.008 25623965

[35] Kim MY, Mauro S, Gévry N, Lis JT, Kraus WL. NAD+-dependent modulation of chromatin structure and transcription by nucleosome binding properties of PARP-1. Cell 2004; 119:803–814. 10.1016/j.cell.2004.11.002 15607977

[36] Guastafierro T, Cecchinelli B, Zampieri M, Reale A, Riggio G, Sthandier O, et al CCCTC-binding factor activates PARP-1 affecting DNA methylation machinery. J. Biol. Chem. 2008; 283:21873–21880. 10.1074/jbc.M801170200 18539602 PMC2494936

[37] Hassa PO, Haenni SS, Buerki C, Meier NI, Lane WS, Owen H, et al Acetylation of poly(ADP-ribose) polymerase-1 by p300/CREB-binding protein regulates coactivation of NF-kappaB-dependent transcription. J. Biol. Chem. 2005; 280:40450–40464. 10.1074/jbc.M507553200 16204234

[38] Mehrotra P, Riley JP, Patel R, Li F, Voss L, Goenka S. PARP-14 functions as a transcriptional switch for Stat6-dependent gene activation. J. Biol. Chem. 2011; 286:1767–1776. 10.1074/jbc.M110.157768 21081493 PMC3023471

[39] Hassa PO, Buerki C, Lombardi C, Imhof R, Hottiger MO. Transcriptional coactivation of nuclear factor-kappaB-dependent gene expression by p300 is regulated by poly(ADP)-ribose polymerase-1. J. Biol. Chem. 2003; 278:45145–45153. 10.1074/jbc.M307957200 12960163

[40] Verdone L, Caserta M, Di Mauro E. Role of histone acetylation in the control of gene expression. Biochem. Cell. Biol. 2005; 83:344–353. 10.1139/o05-041 15959560

[41] Agricola E, Verdone L, Di Mauro E, Caserta M. H4 acetylation does not replace H3 acetylation in chromatin remodelling and transcription activation of Adr1-dependent genes. Mol. Microbiol 2006; 62: 1433–1446. 10.1111/j.1365-2958.2006.05451.x 17121596

[42] de Murcia G, Huletsky A, Lamarre D, Gaudreau A, Pouyet J, Daune M, et al Modulation of chromatin superstructure induced by poly(ADP-ribose) synthesis and degradation. J. Biol. Chem. 1986; 261: 7011–7017. 3084493

[43] Hottiger O. ADP-ribosylation of histones by ARTD1: an additional module of the histone code? FEBS Lett 2011; 585:1595–1599. 10.1016/j.febslet.2011.03.031 21420964

[44] Scobie KN, Damez-Werno D, Sun H, Shao N, Gancarz A, Panganiban CH, et al Essential role of poly(ADP-ribosyl)ation in cocaine action. Proc. Natl. Acad. Sci. USA 2014; 111:2005–2010. 10.1073/pnas.1319703111 24449909 PMC3918779

[45] Hazzalin CA, Mahadevan LC. Dynamic acetylation of all lysine 4-methylated histone H3 in the mouse nucleus: analysis at c-fos and c-jun. PLoS Biol. 2005; 3:e393 10.1371/journal.pbio.0030393 16262446 PMC1278937

[46] Clayton AL, Hazzalin CA, Mahadevan LC. Enhanced histone acetylation and transcription: a dynamic perspective. Mol. Cell 2006; 23:289–296. 10.1016/j.molcel.2006.06.017 16885019

