# Supplementary figures and images for: Poly(ADP-Ribosyl)ation Affects Histone Acetylation and Transcription
Source: PLoS One. 2015 Dec 4;10(12):e0144287. doi: 10.1371/journal.pone.0144287 (PMC4670112; doi:10.1371/journal.pone.0144287)

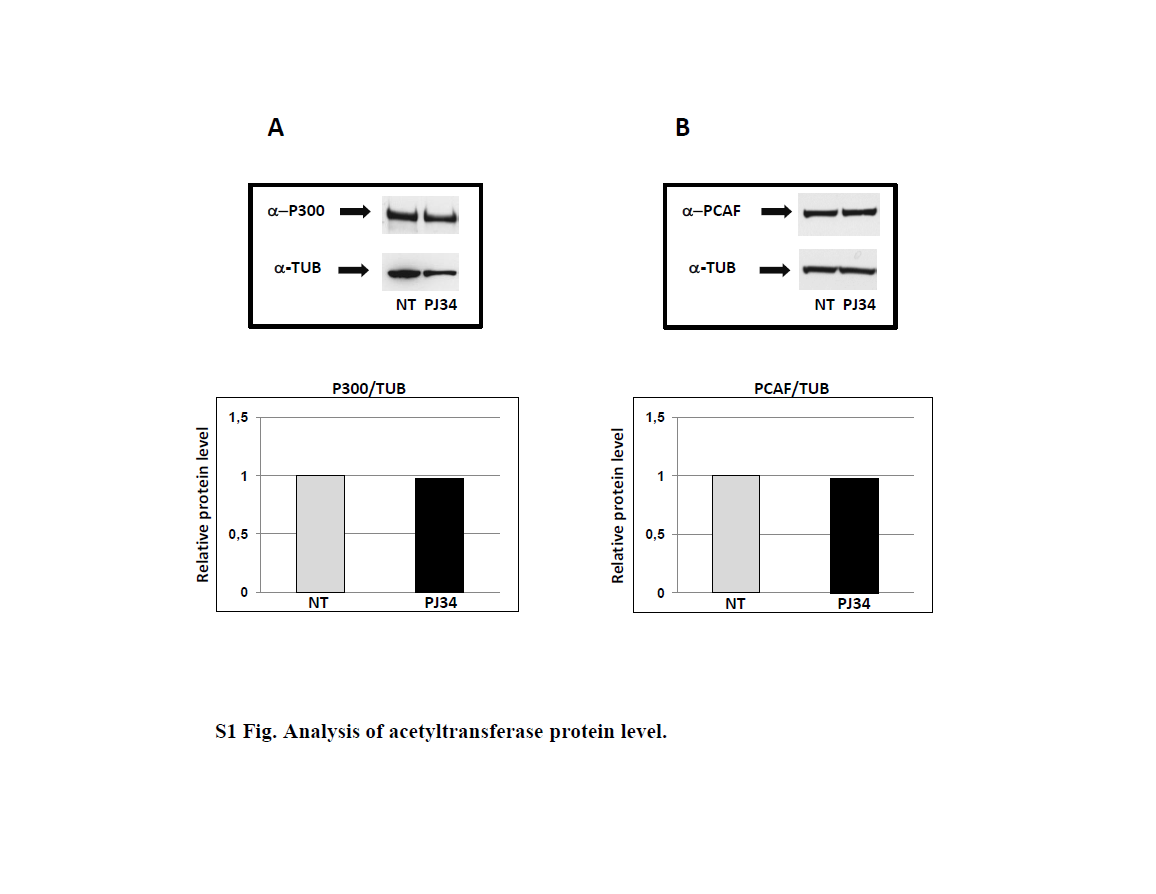

Supplement: S1 Fig — Western blot analysis of p300 (A) and PCAF (B) in extracts from cells treated with PJ34 for 3 h relative to untreated cells (NT). Histograms indicate p300 and PCAF protein level, normalized with α tubulin, from cells treated with PJ34 for 3 h (black) relative to untreated cells (NT, grey). Antibodies used were: mouse monoclonal anti-KAT3B/p300 CT (Millipore, 05–257), rabbit polyclonal anti-KAT2B/PCAF (abcam, ab12188), and rat monoclonal antibody anti-α tubulin (Santa Cruz Biotechnology, sc-53030). (TIF) [file pone.0144287.s001.tif]

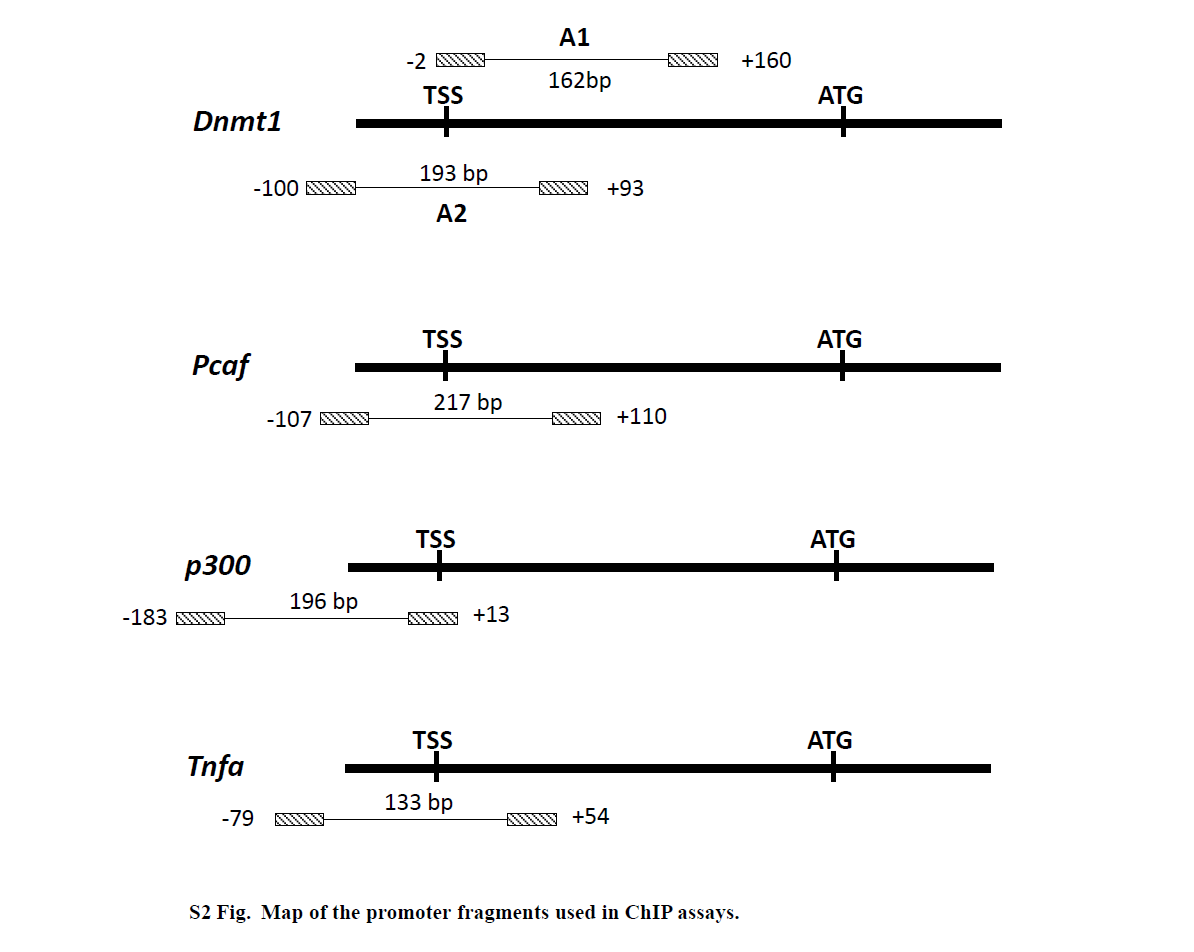

Supplement: S2 Fig — For each gene the TSS (Transcriptional Start Site) and the ATG are indicated. (TIF) [file pone.0144287.s002.tif]

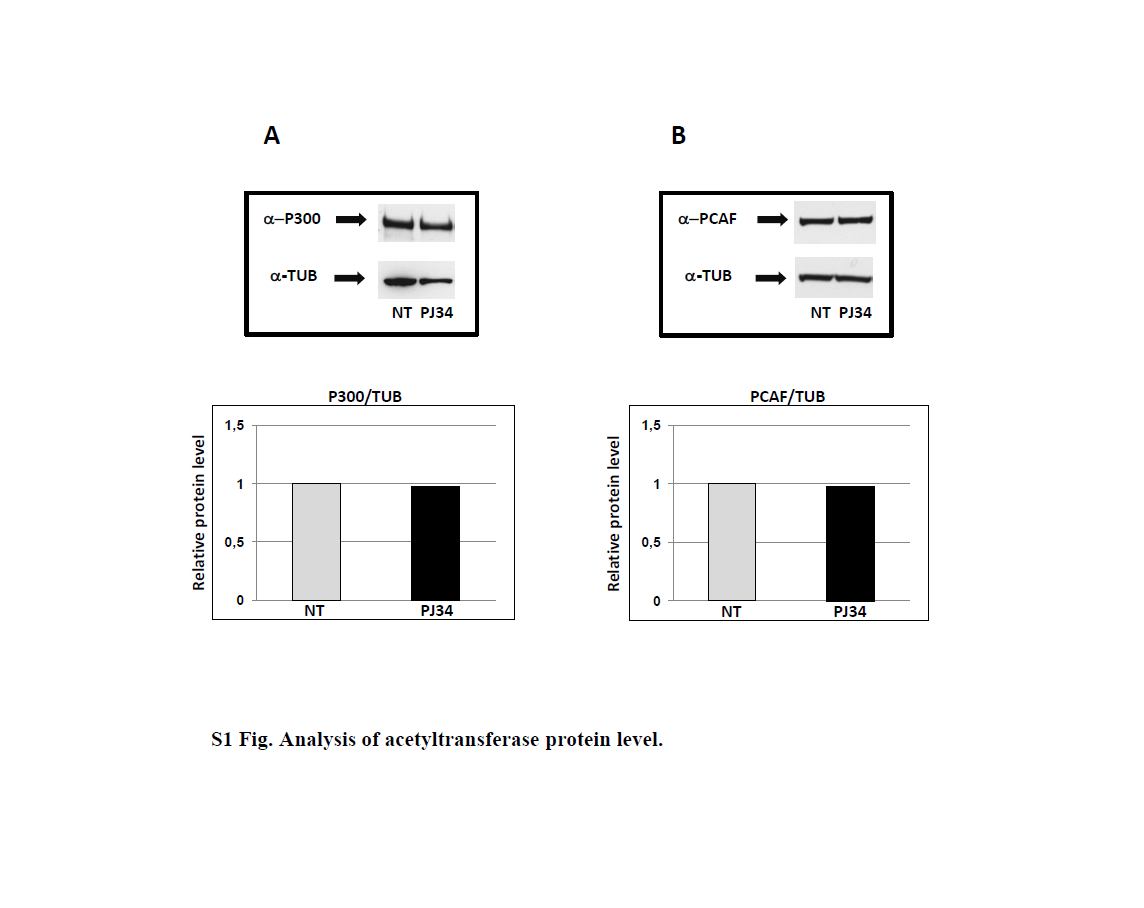

Supplement: S3 Fig — (A) qRT-PCR of Parg mRNA level from NIH3T3 cells treated with PJ34 for the indicated times, relative to untreated cells. The mRNA values were normalised to the mean expression of two housekeeping genes, Gusb and Hprt1. Taqman probes were: Mm00449466_m1 for Parg, Mm00446956_m1 for Gusb, and Mm00446968_m1 for Hprt1. qRT-PCR conditions were as described in Ciccarone et al., Plos One 2008. Error bars indicate the standard deviation of data obtained from three independent experiments. (B) Western blot of whole cell extracts from the same cells as in (A) run on an 8% SDS-PAGE, and probed with anti-PARG (Santa Cruz sc-21480), or anti-PAR, or anti-Tubulin antibodies. (TIF) [file pone.0144287.s003.tif]

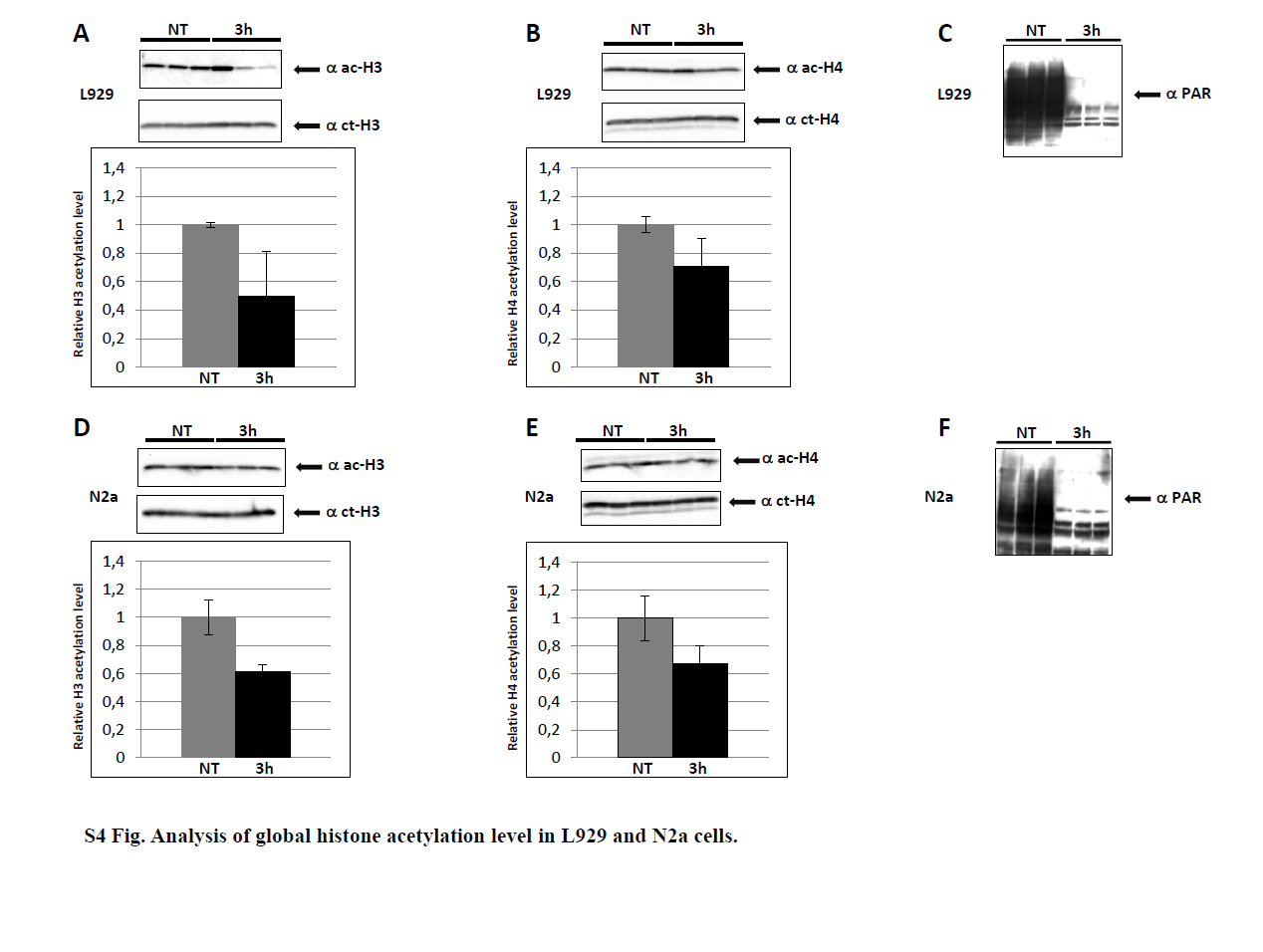

Supplement: S4 Fig — (A) Western blot of whole cell extracts from L929 cells treated with the inhibitor PJ34 (5 M) for 3 h relative to untreated cells (NT, value ~1), run on a 15% SDS-PAGE, probed with anti-acetyl-histone H3 or anti-C-terminal of H3 antibodies to measure the relative level of H3 acetylation. Error bars indicate the standard deviation of data obtained from the three independent experiments. (B) Same as in (A) but hybridisation was performed with anti-acetyl-histone H4 or anti-C-terminal of H4 antibodies to measure the relative level of H4 acetylation. (C) The same extracts used in panels A and B were run on an 8% SDS-PAGE and probed with anti-PAR antibody to visualize ADP-ribose polymers level. Panels D, E and F: same as in A, B and C, respectively but the analysis was done with whole cell extracts from neuroblastoma N2a cells. (TIF) [file pone.0144287.s004.tif]

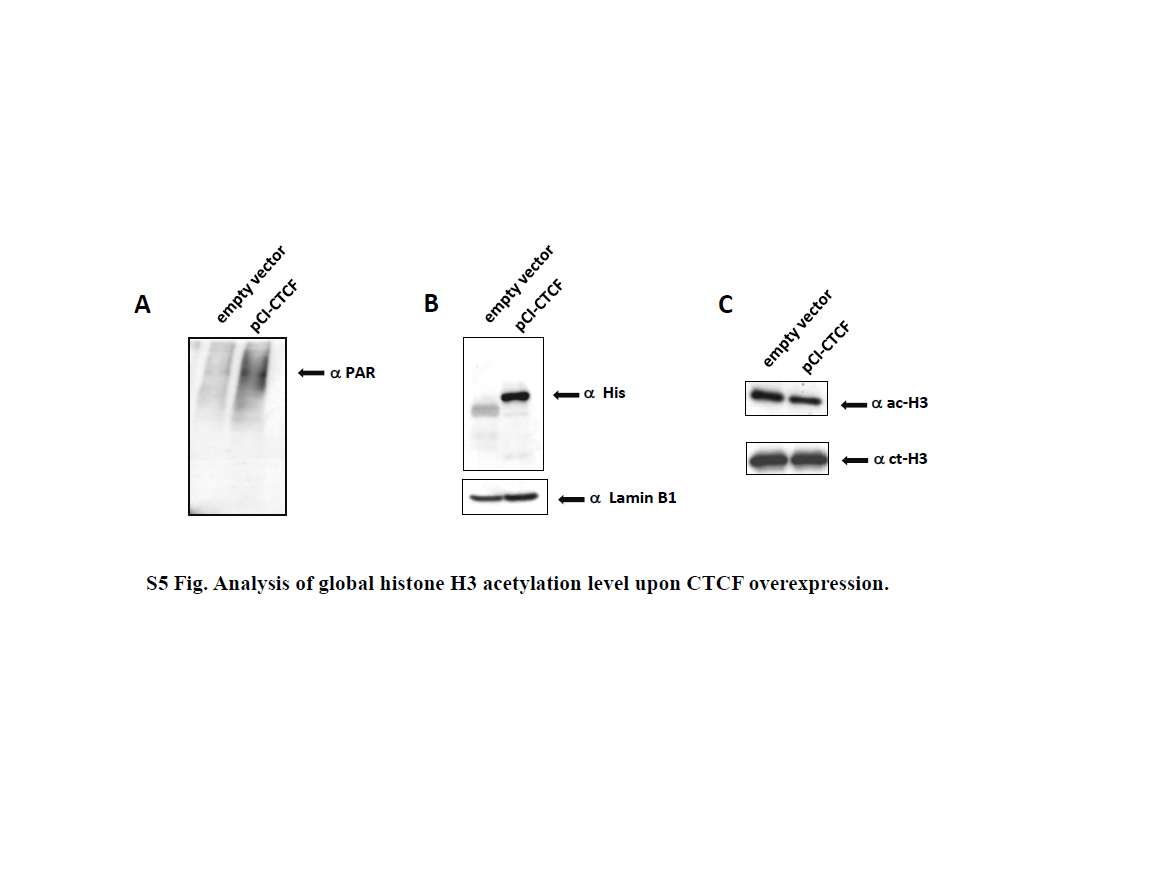

Supplement: S5 Fig — (A) Western blot of total proteins from cells transfected with empty vector (pCI) or His-tagged recombinant CTCF (pCI-CTCF-His, Guastafierro et al., J. Biol. Chem 2008) run on an 8% SDS-PAGE, and probed with anti-PAR antibody. (B) Same as in (A) but hybridisation was performed with anti-His antibody to visualize CTCF, and with anti-Lamin B1, as protein loading control. (C) The same extracts used in panel A and B were run on a 15% SDS-PAGE, and probed with anti-acetyl-histone H3 and anti-C-terminal of H3 antibodies. (TIF) [file pone.0144287.s005.tif]

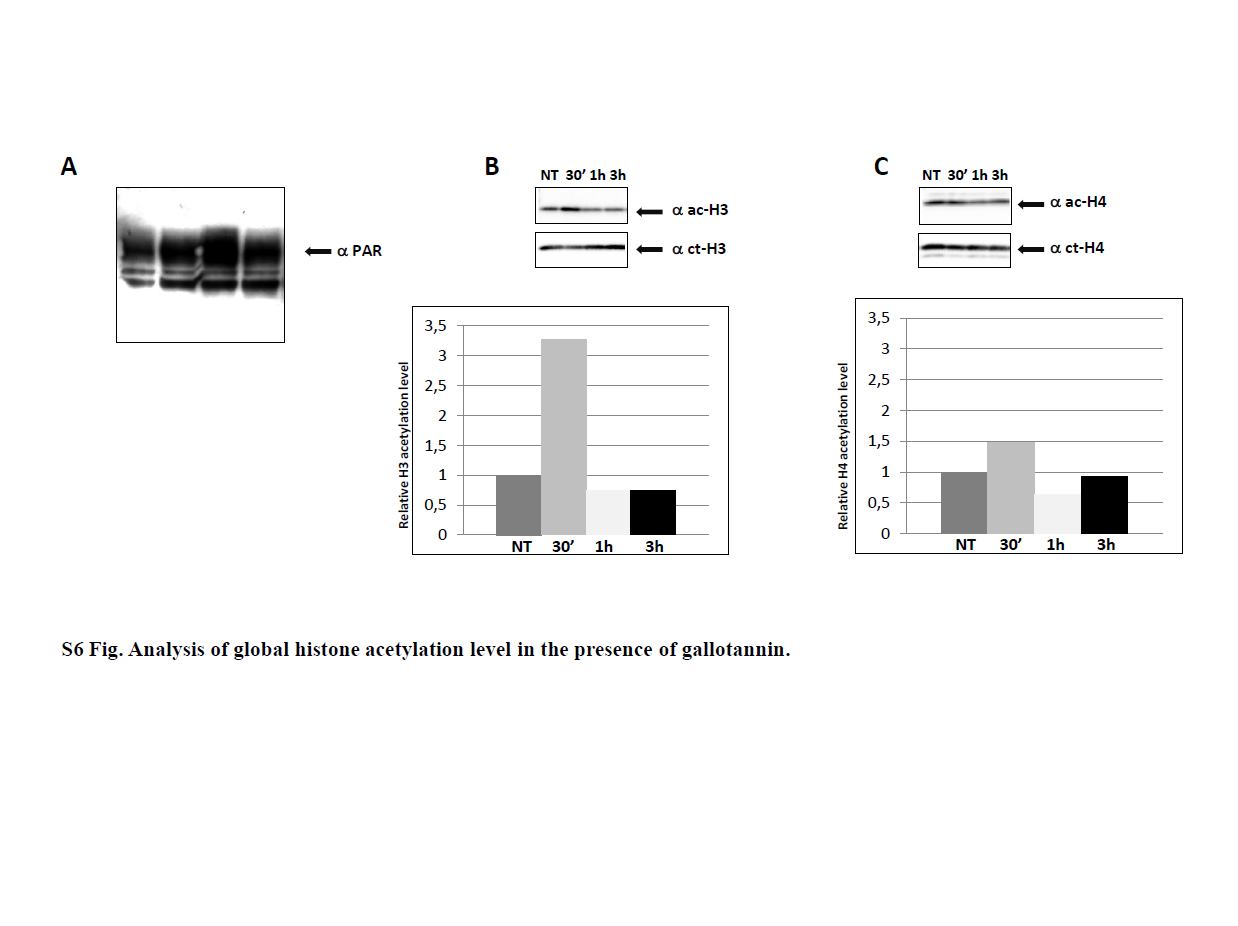

Supplement: S6 Fig — (A) Western blot of whole cell extracts from NIH 3T3 cells treated with the PARG inhibitor gallotannin (Tannic Acid, Sigma) (30 M) for the indicated times relative to untreated cells (NT) run on an 8% SDS-PAGE, and probed with anti-PAR antibody to visualize ADP-ribose polymers. (B) The same extracts as in (A) were run on a 15% SDS-PAGE and probed with anti-acetyl-histone H3 or anti-C-terminal of H3 antibodies to measure the relative level of H3 acetylation. (C) Same as in (B) but hybridisation was performed with anti-acetyl-histone H4 or anti-C-terminal of H4 antibodies to measure the relative level of H4 acetylation. (TIF) [file pone.0144287.s006.tif]
